# The application and impact of artificial intelligence technology in graphic design: A critical interpretive synthesis

**DOI:** 10.1016/j.heliyon.2024.e40037

**Published:** 2024-11-04

**Authors:** Hong Li, Tao Xue, Aijia Zhang, Xuexing Luo, Lingqi Kong, Guanghui Huang

**Affiliations:** a Faculty of Humanities and Arts, Macau University of Science and Technology, Avenida Wai Long, Taipa, Macau, 999078, China; b Zhuhai M.U.S.T. Science and Technology Research Institute, Zhuhai, Guangdong, China

**Keywords:** Atificial intelligence, Graphic design, Machine learning, Computer vision, Visual communication design, Systematic review

## Abstract

In the field of graphic design, the application of Artificial Intelligence (AI) is reshaping the design process. This study employs the Critical Interpretive Synthesis (CIS) approach to explore the impacts and challenges of AI on graphic design. Through a comprehensive review of 33 papers, this research reveals four research paradigms of AI in graphic design: Artificial Intelligence Driven Design Automation and Generation (AIDAG), Artificial Intelligence Assisted Graphic Design and Image Processing (AGDIP), Artificial Intelligence in Art and Creative Design Processes (AACDP), and Artificial Intelligence Enhanced Visual Attention and Emotional Response Modeling (AVERM). These paradigms demonstrate the multidimensional role of AI in design, ranging from automation to emotional interaction. The findings suggest that AI serves a dual role as both a design tool and a medium for innovation. AI not only enhances the automation and efficiency of the design process but also fosters designers' creative thinking and understanding of users' emotional needs. This study also proposes a path for the application of the four paradigms in the graphic design process, providing effective design ideas for future design workflows.

## 1 Introduction

In the digital age, with the ongoing development of artificial intelligence technology, the daily work of graphic designers could undergo revolutionary changes through the integration of AI technology, impacting everything from conceptual thinking to visual expression [1]. While traditional design methods may no longer meet people's current needs, with AI technology support, graphic designers can accurately predict user demands and understand their preferences, creating personalized and appealing visual works [2]. For example, Netflix uses AI technology to analyze user viewing patterns and customise personalized covers, increasing user click-through rates [3]. The aim of this paper is to provide a comprehensive examination of the current state of AI applications in graphic design, discuss the challenges faced and future research directions, and focus on the main paradigms and their impact on graphic design arising from AI technology's rapid development. Artificial Intelligence (AI) is a branch of computer science that aims to create machines or software capable of performing tasks that require human intelligence [4]. Graphic Design (GD) is the art and practice of visual communication and aesthetic expression [5]. It uses visual content to communicate, construct, and solve problems, primarily focusing on compositional elements and spatial layout [6].With the development of technologies such as machine learning and computer vision, emerging automation tools are capable of processing vast amounts of data and generating innovative designs, even mimicking and enhancing human creativity, bringing fundamental changes to the work of graphic designers [7].

Since 2011, the application of genetic algorithms in vector graphics has initiated early attempts of AI in the expression of graphic design [8]. By 2015, semi-automated design assistance tools emerged, capable of analyzing a vast number of graphic elements [9]. Especially demonstrating great potential in the color analysis of LOGO design [10]. Machines capable of mimicking based on painting features appeared simultaneously, predicting and analyzing artists' styles and genres [11]. There were also studies exploring the relationship between texture and aesthetic perception by extracting texture image features, predicting human aesthetic preferences towards different visual textures [12]. By 2016, the use of autoencoders to evaluate the aesthetics of photos by extracting image features had been developed [13], and machine learning was utilized to quantify design principles, paving a new path for AI to evaluate aesthetics [[14], [15], [16]]. In 2019, data mining enhanced design creativity [17], quality [18], and efficiency [19], as well as alleviated the burden of design [20]. In 2020, the applications of real-time augmented reality [21] and digital image [22] technology in graphic design attracted attention. Entering 2023, research trends have become more specific, focusing on using AI assistance tools to help designers understand graphic design style trends [23]. For example, Wang and others proposed a rattan pattern design method that utilizes deep learning and image recognition models, capable of creatively generating patterns based on user emotional preferences [24]. While AI brings opportunities to graphic design, it also faces challenges [25], such as how to maintain personality and creativity while achieving or surpassing human levels, such as the understanding of design complexity and cultural elements [26].Currently, attempts have been made to enhance the naturalness and detail of graphics through algorithms [27], yet computing complex design prototypes remains a challenge to be overcome [28]. This includes the understanding and simulation of designers' creative thought processes [29] and the flexible application of AI in different design scenarios [30], where research is still insufficient. Simultaneously, how to effectively integrate AI technology with graphic design practice, ensure the originality of the design, and enhance human-computer interaction [31,32], as well as address ethical considerations, is also important [33]. Future research should focus on how to utilize big data to provide creative support, ensure the personalization of design works, and consider cultural differences. This includes exploring collaborative models between AI and designers [34], establishing evaluation criteria [35], and enhancing cultural adaptability [36]. Since current research mainly focuses on the application of AI technology in graphic design practice, it is necessary to delve deeper into the impact of AI on designers' work and clarify the role and future development path of AI in the field of graphic design.

This article employs critical interpretive synthesis (CIS) to reflect on the relationship between artificial intelligence and graphic design. CIS represents a synthetic research approach suitable for wide-ranging topics and complex issues [37], particularly utilized in emerging interdisciplinary fields. This method facilitates the discovery of deeper meanings and relationships through critical interpretation of literature. Regarding the search strategy, a comprehensive retrieval was conducted across five databases: Web of Science, Scopus, ScienceDirect, ProQuest, IEEE Xplore, and EBSCO, combining the keywords "Graphic Design" OR "Visual Communication Design" with "artificial intelligence" OR "Machine Learning" OR "AI" OR "Computer Vision". Through the selection process, duplicates, invalid, and off-topic literature were excluded, ultimately identifying 33 pieces of literature. This review takes a comprehensive approach to systematically categorising and analysing the role of AI in graphic design, highlighting how techniques such as machine learning, deep learning and generative adversarial networks are being used to innovate the design process, enhance user interactions, and address challenges such as cultural appropriateness and emotional engagement, providing a holistic perspective. This broad perspective enables a nuanced understanding of the interplay between AI technologies and graphic design outcomes. Unlike previous reviews, this paper not only lists the applications of AI, but also critically analyses their impact, identifies trends and gaps, and suggests future research directions. The purpose of this review is to examine the current state and challenges directions of AI technology in the field of graphic design, with a particular focus on how AI technologies drive paradigm shifts in graphic design. To achieve this objective, the study will revolve around the following two questions.

1) Why has AI developed so rapidly in the field of graphic design, and what are its main paradigms?
2) What are the current research trends and future directions in this field?

## 2 Methods

### 2.1 Critical interpretive synthesis

This study employs the critical interpretive synthesis (CIS) method for a systematic review and analysis of the literature on the application of artificial intelligence in graphic design. The CIS method enables the integration and summarization of existing literature, analyzing and deconstructing the knowledge system from a critical perspective [38], and revealing the implicit relationships and issues within the literature [39,40]. This approach is particularly suitable for exploring the complexities of artificial intelligence applications in graphic design. Additionally, this study adheres to the ENTREQ reporting guidelines to ensure the transparency of the synthesis process [41].

### 2.2 Search strategy

To comprehensively understand the impact of artificial intelligence on graphic design, we crafted a search string "Graphic Design" OR "Visual Communication Design" AND "Artificial Intelligence" OR "Machine Learning" OR "AI" OR "Computer Vision" to perform an exhaustive search across five databases. The research samples were rigorously screened, with inclusion criteria comprising: (a) studies focused on the application of artificial intelligence in the graphic design process or outcomes; (b) studies employing empirical, theoretical, and case study methodologies, including journal articles and conference papers; (c) articles written in English to ensure the accuracy of content comprehension. Exclusion criteria encompassed: (a) non-peer-reviewed articles, documents solely focusing on pedagogical content, technical reports, patents, tutorials, and books; (b) articles focusing on the application of artificial intelligence outside the field of graphic design; (c) conceptual papers lacking empirical research support or specific case analyses. Preliminary screening was then conducted based on titles and abstracts, with full-text readings focusing on the relevance of research questions [42]. The quality of the literature was assessed according to the Mixed Methods Appraisal Tool (MMAT) [43] with discussions and screenings carried out by two researchers. In case of disagreement, a third researcher was consulted to resolve any differences in opinions regarding content and methodology. To avoid the limitations of previous research, concepts related to graphic design and artificial intelligence (see Table 1) were used as the basis.

**Table 1:** Summary of definitions of search term concepts.

Concept	Definition
Graphic design	Graphic designers use visual elements across various mediums to convey information and concepts, collaborating with other creatives to produce engaging designs [44].
Visual communication design	Visual communication design is a creative process that combines imagery, typography, color, and composition to express emotions and convey information [45].
Artificial intelligence	This field of study focuses on creating computational systems that mimic human perception, cognition, and actions, offering solutions to complex challenges [46].
Machine learning	Machine learning is a field where computers learn from experience to enhance their own performance, akin to human cognitive processes [47].
Computer vision	Computer vision is an area of AI where machines are programmed to process and analyze visual information, enabling them to recognize images, understand scenes, and perform tasks that require visual understanding, similar to how humans see and interpret the world [48].

### 2.3 Data extraction

**Fig. 1:**
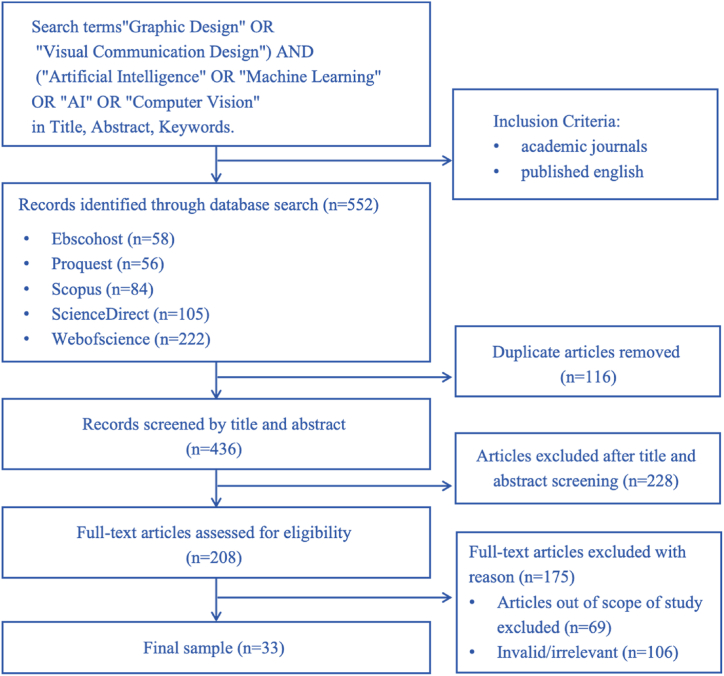
Flowchart of systematic literature screening.

Following a comprehensive search from 2010 to October 2023, a total of 552 pieces of literature were identified. Titles and abstracts were screened, and the search results were saved in Endnote 20. A preliminary assessment was conducted based on the inclusion/exclusion criteria, leaving the remaining documents considered potentially suitable. After full-text reading to evaluate and confirm their relevance to the research criteria, ultimately, 33 pieces of literature that fit the scope of this study were included (details can be seen in Fig. 1 and Table 2).

**Table 2:** Literature included in the study n = 33.

Author(s)	Title	Journals
[49]	Automatic layout algorithm for graphic language in visual communication design	International Journal of Advanced Computer Science and Applications
[50]	AnimateSVG: autonomous creation and aesthetics evaluation of scalable vector graphics animations for the case of brand logos	Proceedings of the AAAI Conference on Artificial Intelligence
[51]	Design of graphic design assistant system based on artificial intelligence	International Journal of Information Technologies and Systems Approach
[52]	PLay: parametrically conditioned layout generation using latent diffusion	ICML'23: Proceedings of the 40th International Conference on Machine Learning
[53]	Predicting visual attention in graphic design documents	IEEE Transactions on Multimedia
[54]	A study of artificial intelligence-based poster layout design in visual communication	Scientific Programming
[55]	Symbolic model of new media art expression based on artificial intelligence big data	Wireless Communications & Mobile Computing
[56]	Design on intelligent feature graphics based on convolution operation	Mathematics (2227–7390)
[57]	Addressing the use of artificial intelligence tools in the design of visual persuasive discourses	Designs
[58]	Exploration and application of graphic design language based on artificial intelligence visual communication	Wireless Communications & Mobile Computing
[59]	Visual memory neural network for artistic graphic design	Scientific Programming
[60]	Study on the application of visual communication design in APP interface design in the context of deep learning	Computational Intelligence & Neuroscience
[61]	Visual communication design of image multidimensional visualization fusion system based on machine learning	2022 2nd Asia-Pacific Conference on Communications Technology and Computer Science (ACCTCS)
[62]	Image harmonization based on the semantic information of foreground human	Displays
[63]	Artificial intelligence empowered visual communication graphic design	2021 International Conference on Networking Systems of AI (INSAI)
[64]	Perceptual similarity measurement based on generative adversarial neural networks in graphics design	Applied Soft Computing
[65]	Learning personal style from few examples	DIS '21: Proceedings of the 2021 ACM Designing Interactive Systems Conference
[66]	Design of semantic-based colorization of graphical user interface through conditional generative adversarial nets	International Journal of Human-Computer Interaction
[67]	Research on the regenerated design of blue calico based on computer image processing	Culture and Computing: 8th International Conference
[68]	Prediction of the emotion responses to poster designs based on graphical features: A machine learning-driven approach	Archives of Design Research
[69]	Analysis of computer graphic image design and visual communication design	In 2020 5th International Conference on Mechanical, Control and Computer Engineering
[70]	Real-time AR technology assisted high-resolution image processing and its graphic design application	IEEE Access
[71]	Research and extraction on intelligent generation rules of posters in graphic design	Cross-Cultural Design. Methods, Tools and User Experience: 11th International Conference, CCD 2019
[72]	Evolving stencils for typefaces: combining machine learning, user's preferences and novelty	Complexity
[73]	Context-aware asset search for graphic design	IEEE Transactions on Visualization and Computer Graphics
[74]	Application research of digital image technology in graphic design	Journal of Visual Communication and Image Representation
[75]	Usage of artificial intelligence in today's graphic design	Online Journal of Art & Design
[76]	What characterizes personalities of graphic designs?	ACM Transactions on Graphics
[77]	Computational aesthetic evaluation of logos	ACM Transactions on Applied Perception
[78]	Unified photo enhancement by discovering aesthetic communities from flickr	IEEE transactions on Image Processing
[79]	Semi-automatic color analysis for brand logos	Color Research & Application
[80]	Exploring the space of abstract textures by principles and random sampling	Journal of Mathematical Imaging and Vision
[81]	Automatic and interactive evolution of vector graphics images with genetic algorithms	Visual Computer

### 2.4 Quality assessment

The screening process for this research was independently carried out by the first author. and subsequently reviewed by the second author. The mixed methods appraisal tool (MMAT) was employed for quality assessment, and in cases of differing scores, disagreements were resolved through discussion. The MMAT is designed specifically for assessing the quality of both mixed methods and single-method research designs [43]. The assessment tool consists of 15 questions, categorising each study into weak (≤0.50), moderately weak (0.51–0.65), moderately strong (0.66–0.79), or strong (≥0.80). This scoring system has been utilized in multiple review articles [82,83].

### 2.5 Data Analysis:conducting an interpretive synthesis

Critical interpretive synthesis belongs to meta-ethnography, an interpretive synthesis approach [38]. Through iterative analysis and thematic coding, literature was cross-compared using Excel grids to reveal similarities and contradictions between studies [84]. This article employs the four steps of critical interpretive synthesis [37,85] for planned analysis (see Table 3), detailed as follows:

**Table 3:** Interpretive synthesis steps (based on Flemming, 2010).

Phase of cis	Processes involved
Phase 1: literature comprehension	The selected papers were read carefully, and key features were extracted and recorded in a table.
Phase 2: study association	Categorising themes, translating themes across studies and refining content.
Phase 3: translation analysis	Interpret evidence holistically and translate data into new conceptual forms.
Phase 4: synthetic expression	Analysing synthetic constructs and reflecting on inter-construct relationships.

Literature comprehension: At the beginning of the analysis, we meticulously read the 33 included pieces of literature. Starting from the research question, we recorded the background and relevant characteristics of each study, endeavoring to identify methods and content related to the question [86]. The data extracted from the articles include journal publishers, publication years, research backgrounds, research objectives, research questions, design contents, and artificial intelligence application technologies. All selected studies were coded individually to extract core concepts and themes for a comprehensive understanding of existing research [87].

Study association: Concepts and elements within the literature that could be categorized into another study to simplify all research content and backgrounds.

Translational analysis: Elements were translated and categorized, interpreting content as a whole and converting data into new conceptual forms or synthetic structures, known as Reciprocal Translation Analysis (RTA) [37]. Through RTA, content can be transformed into a new conceptual form.

Synthetic expression: integrating all research content to form a coherent structure. By conducting a detailed analysis of the foundational argument formed in the first three steps, a new conceptual framework is synthesized [38]. During this process, the frequency distribution and categorization of synthesized materials are reflected upon to consider the relationships within their internal structure [85].

## 3 Findings

### 3.1 Reference data extraction:description of studies

In the 33 included studies, authors primarily hail from China, the United States, and South Korea, with China producing a considerable number of publications on the topic. This may be related to the unique context and structure of the Chinese academic community, where there's a compulsory uniformity in the terminology of graphic design. While this practice aids in maintaining communication efficiency within the discipline, it might also inhibit the exploration of conceptual boundaries and the integration of interdisciplinary perspectives. In terms of research methodologies, experimental research was conducted 25 times, mixed methods research 3 times, qualitative research 3 times, and case studies 2 times, as detailed in Table 4. The widespread use of experimental research might be attributed to the field's emphasis on empirical validation of technology and applications. We meticulously reviewed the selected literature, focusing on their research objectives, questions, design applications, and key elements and main outcomes of AI technology, such as the types of AI technologies, applications in graphic design, and research findings, with detailed information provided in Appendix 1. Based on the MMAT quality assessment criteria, the included literature underwent a quality evaluation (see Appendix 2). The evaluation results revealed that 14 studies fully met the quality standards (100 %), 7 studies achieved over 80 % of the quality standards, 10 studies reached over 60 % of the quality criteria, and only 1 study met 40 % of the quality standards. This indicates that many of the studies are methodologically sound, providing valid insights and evidence for this research.

**Table 4:** Frequency tables for country region, publisher, and research method (n = 33).

Date	Home countries	Publisher	Case study	Experiment research	Mixed methods	Qualitative	Total
2012	Canada	Springer		1			1
2015	New Zealand	John Wiley and Sons Inc.	1				2
	Spain	Springer		1			
2016	China	Institute of Electrical and Electronics Engineers Inc.		1			1
2017	China	Association for Computing Machinery		1			1
2018	China	Association for Computing Machinery			1		2
	Turkey	Online Journal of Art & Design	1				
2019	China	Academic Press Inc.		1			4
		Springer		1			
	USA	IEEE		1			
	Portugal	Hindawi		1			
2020	China	IEEE				1	5
		Institute of Electrical and Electronics Engineers Inc.		1			
		Springer		1			
	South Korea	Korean Society of Design Science			1		
		Taylor & Francis		1			
2021	China	Elsevier		1			3
		IEEE		1			
	USA	Association for Computing Machinery			1		
2022	China	Elsevier		1			9
		Hindawi		3			
		IEEE				1	
		MDPI		3			
	Mexico	MDPI				1	
2023	China	IGI Global		1			5
		JMLR.org		1			
		Science and Information (SAI) Organization		1			
	Germany	AAAI Press		1			
	USA	Institute of Electrical and Electronics Engineers Inc.		1			
total			2	25	3	3	33

**Fig. 2:**
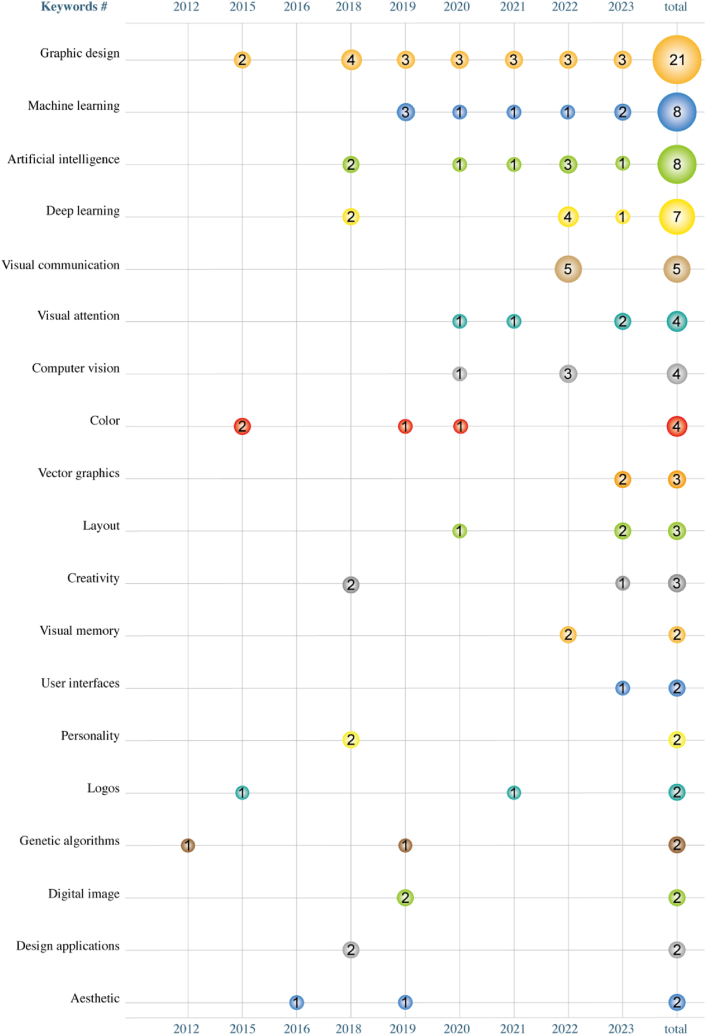
Frequency distribution of keywords in the top 19 papers.

Through frequency analysis of the keywords in the included literature (see Fig. 2), we found that “graphic design” appeared 21 times, “machine learning” and “artificial intelligence” each appeared 8 times, demonstrating a strong interest in the design community for efficiency improvements, automation, and AI applications. Additionally, “deep learning” appeared 7 times, emphasizing the potential in image processing and pattern recognition. “visual communication” occurred 5 times, possibly reflecting an emphasis on using AI to enhance the effectiveness of information transmission. “visual attention,” “computer vision,” and “color” each appeared 4 times, indicating a focus on using AI to capture visual attention, perform computer vision analysis, and apply color theory. “vector graphics,” “layout,” and “creativity” each appeared 3 times, highlighting the emphasis on the creative layout of graphic elements and the pursuit of originality in design. Lastly, the lower frequency of keywords such as “visual memory,” “aesthetic,” “personality” might indicate that, despite not being extensively discussed, there is a certain demand for these areas. This trend suggests an emerging interest in how design connects with audience perception and personalized experiences.

### 3.2 Research correlation: integration of graphic design & AI technology

By encoding the literature, we systematically integrated key elements of AI technology and graphic design applications, details of which can be found in Appendices 1, with some high-frequency summaries presented in Table 5. From 2012 to 2015, the introduction of "evolutionary art" [81] and "genetic algorithms" [88] showcased an interest in generative and iterative design. Discussions on how AI began affecting color selection and brand strategies through "color recognition" and "data mining algorithms" emerged [79], with machine learning techniques being utilized to imitate natural colors and textures, enhancing the aesthetics of digital graphics [80]. The trend towards automation was reinforced from 2016 to 2018, especially with deep learning technologies like "aesthetic communities" and "photo enhancement framework" [78] being widely applied to design tasks [76,[89], [90], [91]], and the first appearance of community aesthetics-based photo enhancement models [78]. Deep learning was employed to provide "personalized" scoring for graphic design works, predicting the works' personality traits [76]. From 2019 to 2020, intelligent generation and optimization of design elements such as “typography,” “Color,” and “Visual hierarchy” reached new heights [71]. Entering 2021 to 2022, AI-assisted graphic design showcased personalization and innovation through aesthetic rules of form, repetitive patterns, and customized visual examples [63,92], like the new enhanced deep learning-based automated historical image colorization in wireless network-enabled visual communication (EDL-AHIC) technology developed for the automatic coloring of grayscale cultural images [60]. By 2023, innovations in emotional expression with “Sentiment-specific word embedding” and “Conditional generative adversarial nets” emerged [51]. A hybridized grid and content-based automatic layout (HGC-AL) algorithm could automate layout balancing, enhancing the potential for design precision and aesthetics [49]. AI technology has become a key driving force for future research, discussed in detail in the following two sections.

**Table 5:** A partial frequency table of key elements of AI technology and graphic design.

Factors	2012	2015	2016	2017	2018	2019	2020	2021	2022	2023	total
AI technology											
Machine learning					1	3	2		1	1	8
Deep learning								1	6		7
Convolutional neural network					1			1	1	1	4
Conditional generative adversarial nets							1			1	2
Image processing		1					1		1		3
Sentiment-specific word embedding							1			1	2
Content of graphic design											
Color		1				2	1		1		5
Visual communication design							1		3		4
layout						1	1		1		3
Logo design		1						1			2
Packaging design					1		1				2
Textual design concept							1			1	2
Usage frequency							1			1	2

Graphic design component: Firstly, Color, as the most basic and crucial element in design [68], combined with deep learning, can achieve more accurate personalized color matching, speeding up design efficiency and information communication [51]. Secondly, through deep learning and image processing, AI can assist visual communication design in conveying information more clearly, promoting personalized and innovative expression [55]. In terms of Layout, using generative adversarial networks to automatically generate design layouts optimized information hierarchy, attractiveness, and integrity [53]. In computer graphics, particularly convolutional neural networks, innovative methods for image creation, processing, and rendering were provided, enabling multidimensional visualization and high-resolution graphics. Additionally, the application of machine learning in logo design and aesthetic evaluation [50], and balancing aesthetics with functionality in Packaging Design [69], displayed its significant potential in enhancing design quality and innovation. Lastly, in textual design concepts, AI embeds design concepts into vector spaces, enabling the generation of design elements based on emotions and concepts, like color combinations [51,66].

Artificial intelligence technology component: The role of AI technology gradually shifted from simple image processing and texture synthesis to a key driver for design automation and intelligent interpretation. Machine learning, as a core tool for enhancing creative outcomes, its applications in pattern recognition and sentiment analysis allow computers to recognize patterns and make predictions or decisions [69]. Deep learning, a subset of machine learning, is used for image processing, automatic layout generation, image classification, and aesthetic evaluation tasks [55,60]. Additionally, Convolutional neural networks, a network structure in deep learning, are well-suited for processing visual images and widely adopted for their high accuracy in image recognition and visual tasks [65]. Conditional generative adversarial nets, capable of generating color sets that conform to specific design semantics, showcased potential in design automation and intelligent interpretation [64]. Image Processing, a technique for improving or extracting information from images, plays a crucial role in graphic design generation, pattern reconstruction, and color analysis [56]. Computer vision, capable of recognizing and processing visual content in images, can be used for image colorization and high-resolution image processing [74]. Lastly, Genetic algorithms, heuristic search algorithms that mimic the process of natural selection, have been applied in font design and the evolution of vector graphics [81].

**Fig. 3:**
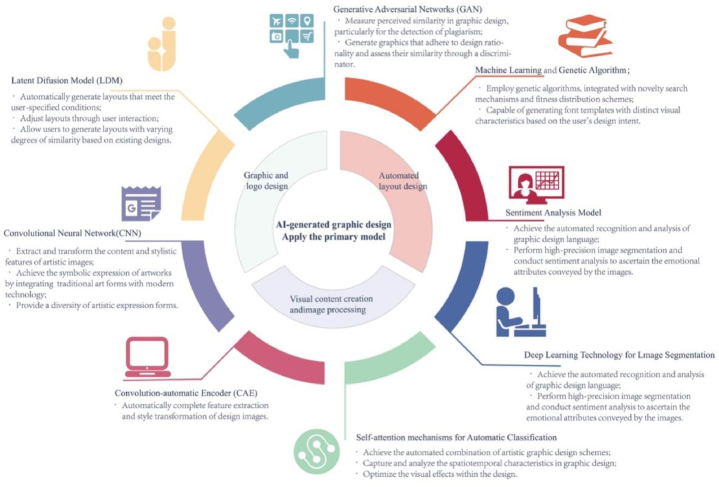
A summary of the latest models and frameworks for generating graphic designs with artificial intelligence.

In the current field of visual communication design, Table 6 and Fig. 3 summarise the AI models proposed in several studies for different design domains. Specifically, Table 6 covers a range of application areas including automated graphic layout design, brand logo animation, graphic design assistance systems, interface design, new media art, and cultural creative product design.

**Table 6:** Recent applications of AI techniques in visual communication design: summary of model characteristics and design implications (2019–2023).

Author(s)	Application area	Model	Model characteristics	Impact on Design
(Liao & Hu, 2023) [49]	Automated graphic layout design	Hybridized grid and content-based automatic layout (HGC-AL)	• Integrating grid and content-driven layouts for responsive fluid grid arrangements; • Automated layout transitions, adhering to design principles and constraints.	Enhances aesthetics and visual appeal, enables more efficient design processes, and automates content arrangement based on design constraints.
(Mateja et al., 2023) [50]	Brand logo animation	Ensemble tree model optimization tool (ENTMOOT)	• Generate SVG animations for brand logos; • Conduct aesthetic evaluations of the generated animations.	Demonstrates AI's potential to create aesthetically pleasing logo animations, introduces innovative network architecture.
(Liu, 2023) [51]	Graphic design assistance system	AI-based graphic design assistant system; convolution-automatic encoder (CAE)	• Achieve image recognition, generation, and aesthetic analysis of geometric elements in graphic design. • Automatically complete feature extraction and style transformation of design images.	Enhances design efficiency through automated and intelligent design tools, expanding the creative space for designers.
(Cheng et al., 2023) [52]	Layout design	Latent Diffusion Model (LDM)	• Automatically generate layouts that meet the user-specified conditions; • Adjust layouts through user interaction; • Allow users to generate layouts with varying degrees of similarity based on existing designs.	Provides interactive and controllable layout generation processes, bringing novel interaction experiences to professional layout design workflows.
(Chakraborty et al., 2023) [53]	Design and user experience	Attention on graphic designs (AGD)	• Predicting the distribution of visual attention and browsing pathways of users within graphic design documents; • Providing an in-depth understanding of document layout and content.	Offers a new method to understand and predict how users visually interact with graphic design documents, including web pages and posters.
(Huo & Wang, 2022) [54]	Poster layout design	Lenet convolutional neural network (CNN); spatial transformer network (STN)	• Identify and locate layout elements in posters; • Optimize initial layout templates to generate various design schemes that meet aesthetic standards.	Employs deep learning technology to automate the poster layout process, including element recognition, classification, and layout optimization.
(Lee, 2022) [55]	New media art	New media art symbolism model	• Extract and transform the content and stylistic features of artistic images; • Achieve the symbolic expression of artworks by integrating traditional art forms with modern technology; • Provide a diversity of artistic expression forms.	Provides a new method to simulate and understand new media art expressions, validated through simulation experiments.
(Li & Tang, 2022) [56]	Intelligent image processing	Image feature extracted model based on convolution operation	• Extract image brightness features from traditional engraving graphics; • Process the image brightness features to simplify the image feature extraction process; • Utilize convolution operations to enhance the accuracy of feature extraction.	Proposes a convolution-based image feature extraction model that effectively extracts features of traditional carving graphics for intelligent graphic design.
(Ruiz-Arellano et al., 2022) [57]	Visual content creation	Model for persuasive design	• Integrate artificially intelligent-generated images into the design of visually persuasive discourse; • Offer a multi-stage design process.	Integrates AI technology into the design process of persuasive visual discourses, enhancing design efficiency and originality.
(Lu & Huang, 2022) [58]	Intelligent design assistance	Deep learning-based image segmentation and sentiment analysis model	• Achieve the automated recognition and analysis of graphic design language; • Perform high-precision image segmentation and conduct sentiment analysis to ascertain the emotional attributes conveyed by the images.	Enhances the capability of intelligently recognizing graphic design language, providing designers with a new tool to enhance information transmission and emotional expression in design works.
(Zheng, 2022) [59]	Artistic graphic design	Visual memory neural network for artistic graphic design	• Achieve the automated combination of artistic graphic design schemes; • Capture and analyze the spatiotemporal characteristics in graphic design; • Optimize the visual effects within the design.	Proposes a new method to improve the accuracy of scheme reorganization and quantitative evaluation of artistic models in graphic design.
(Luo & Zeng, 2022) [60]	Interface design	Enhanced deep learning-based automated historical image colorization (EDL-AHIC)	• Automatically colorize historical grayscale images; • Extract and integrate both local and global features of the images to generate high-quality colored images.	EDL-AHIC technology surpasses existing techniques in image colorization, contributing to improved quality of visual communication design.
(Ni & Zhang, 2022) [61]	Image visualization	Image multidimensional visualization fusion system	• Multidimensional visualization presentation of images; • Optimization of image processing and feature extraction; • Support for the handling and analysis of complex image data.	Significantly enhances the innovation and implementation efficiency of graphic design and visual communication design, enriching the expressiveness of artworks.
(Lin et al., 2022) [62]	Image synthesis	Human parsing network; image harmonization network	• Achieve precise segmentation of the foreground portrait and adjust its color and style based on the background image; • Generate more realistic composite images while reducing issues of color distortion.	Advances the use of AI technology in image editing and graphic design, improving the realism and visual appeal of synthesized images.
(Qu et al., 2021) [63]	Visual advertising	Intelligent design robot; generative adversarial network (GAN)	• Automatically generate graphic design solutions; • Utilize GAN (generative adversarial network) models to create high-quality designs and optimize the design effects through principle-based discriminators; • Develop intelligent assistant tools based on design principles to enhance the aesthetic quality and efficiency of the design process.	Propels the application of AI technology in visual communication graphic design, enhancing design efficiency and the artistic expression of works.
(Yang, 2021) [64]	Graphic design	Constraint generative adversarial network (GAN)	• Measure perceived similarity in graphic design, particularly for the detection of plagiarism; • Generate graphics that adhere to design rationality and assess their similarity through a discriminator.	Successfully measures perceptual similarity in graphic design through constrained GAN models, offering a new method for detecting plagiarism in artistic works.
(Lin & Martelaro, 2021) [65]	Graphic design	PseudoClient	• Learn personal style from a small set of positive and negative design examples; • Predict whether a new design conforms to the personal style by calculating the similarity between the graphic design samples and the personal style samples.	PseudoClient excels in accurately learning individual styles, showcasing its potential as a foundational tool for future design applications.
(Lee & Cho, 2020) [66]	User interface design	Conditional generative adversarial network (CGAN)	• Generate a suitable color set based on design semantics; • Create color schemes that meet actual design requirements by integrating design semantics such as application features, design concepts, and usage frequency.	Proposes a new method for GUI design color generation that effectively considers design semantics and usability factors, enhancing the practicality and aesthetics of GUI design.
(Wang & Fu, 2020) [67]	Cultural creative product design	Deep convolutional generative adversarial network (DCGAN)	• Generate new indigo batik patterns from random noise; • Integrate modern graphic design with traditional indigo batik patterns to achieve innovative redesign of traditional designs.	Implements the regenerative design of intangible cultural heritage blue calico patterns, demonstrating the feasibility and effectiveness of this technology in pattern innovation.
(Kim & Suk, 2020) [68]	Graphic design	Machine learning (kknn、svmRadial、C5.0)	• By extracting the graphical features of a poster, predict the audience's emotional response to the poster; • Evaluate and optimize the graphic design elements to enhance the intended emotional effect.	Demonstrates the effectiveness of machine learning models in predicting emotional assessments, particularly excelling in predicting pleasure and dominance.
(Sheng, 2020) [70]	Graphic design	Maximum entropy model	• The algorithm possesses a broad range of applications, robust detail preservation capabilities, and natural effects; • It supports real-time video enhancement and graphic design, offering strong practical utility.	Promotes the development of innovative visual expression and design methods in graphic design, enhancing the three-dimensional visual effects and visual appeal of design works.
(Martins et al., 2019) [72]	Typeface design	Evolutionary system; fitness function design Interface	• Employ genetic algorithms, integrated with novelty search mechanisms and fitness distribution schemes; • Capable of generating font templates with distinct visual characteristics based on the user's design intent.	Demonstrates how to guide the evolution of typeface templates through user-customized fitness functions, affecting design outcomes and enhancing design diversity and innovation.
(Kovacs et al., 2019) [73]	Graphic design	Context-aware asset search	• Focus on image search and color selection; • Utilize the learned model to rank image search results or provide color recommendations; • Address issues in traditional training methods through novel data collection procedures.	Enhances the practicality and integration of design tools by assessing image and color compatibility in design through crowdsourced data collection and new evaluation methods.

Looking at the research content, studies in the fields of automated graphic layout design and visual advertising design are prominent. AI technology has introduced parametric conditional layout generation and deep learning image segmentation techniques, achieving automation in the design process and significantly reducing design time and costs. In terms of model and method application, generative adversarial networks (GAN) and its variants, as well as convolutional neural networks (CNN) and its variants, are the most common choices. These technologies are not only used for image recognition, generation, and aesthetic analysis but also for predicting the distribution of users' visual attention and achieving multidimensional visualization and image synthesis. Through these technologies, researchers have successfully achieved high-quality visual effect generation and optimization.

The impact on design is also significant. AI technology not only improves the efficiency of design work but also expands the creative space for designers through automated tools. Moreover, by predicting users' visual interactions and optimizing design elements, AI technology helps to enhance the information transmission and emotional expression capabilities of design works. In visual advertising and cultural creative product design, the application of AI has also promoted the integration of traditional design elements with modern technology, providing new possibilities for the innovation of traditional patterns and the improvement of quality in visual communication design.

**Fig. 4:**
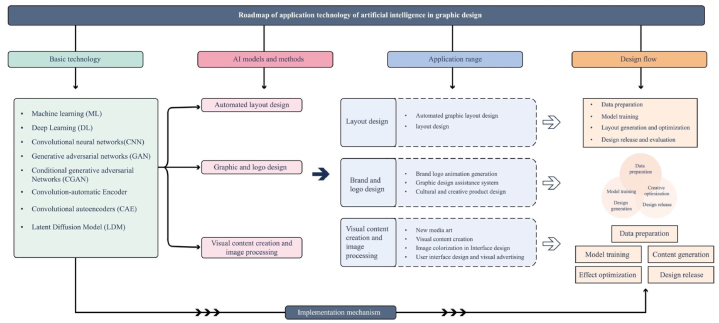
Roadmap of application technology of artificial intelligence in graphic design.

Finally, it was found from the cutting-edge applications of AI in the field of graphic design in recent years that they cover the underlying technologies, AI models and methods, as well as the scope of applications and design processes (as shown in Fig. 4). This technology roadmap for the application of AI in graphic design describes in detail the complete path from the underlying technology to the design process to the implementation mechanisms. At the basic technology level, it covers AI models and methods such as ML, DL, LDM and CNN, which provide powerful algorithmic support for design. The wide range of application areas, including brand logo design, cultural and creative products, and new media art, shows the diversified value of AI technology. In the design process part, a systematic design methodology is formed from data preparation to model training, to automated layout design and optimization, up to design release and evaluation. Such as CNN, GAN, CGAN, CAE, DCGAN and LDM technologies play key roles in the process, which not only improve the automation and personalization of the design, but also enhance the interaction between the design work and the user's emotions through sentiment analysis. Overall, this roadmap shows how AI can help graphic design achieve innovation, improve efficiency, and create more engaging and compelling work.

### 3.3 Translation analysis: four paradigms

**Fig. 5:**
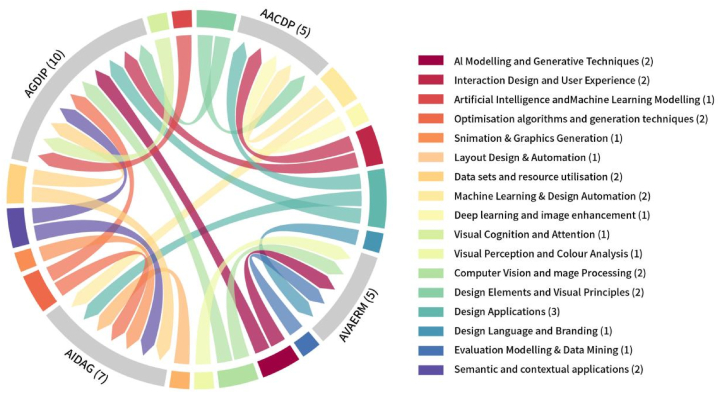
Relationship between the four paradigms and each classification.

Based on the induction and summary of key elements, the process involved a comprehensive consideration of the types of technology, design content, key features, and their application backgrounds, synthesizing a series of detailed categories and themes. This synthesis resulted in the formation of four prominent AI application paradigms, namely, AI-driven design automation and generation (AIDAG), AI-assisted graphic design and image processing (AGDIP), AI in artistic and creative design processes (AACDP), and AI-enhanced visual attention and emotional response modeling (AVAERM), along with 17 categories. Fig. 5 illustrates the association between paradigms and categories. The emergence of these paradigms is based on the induction of the relationships between themes and categories, as well as their universality and representativeness demonstrated in the literature. Table 7 provide detailed results on the classification of paradigm elements, the number of categories, their temporal distribution, and coding examples.

**Table 7:** Four paradigm time-frequency tables.

Themes	Form	2012	2015	2016	2017	2018	2019	2020	2021	2022	2023	all
AACDP	Machine Learning & Design Automation	4		1		10	8		3	1		27
	Interaction Design and User Experience			1	1		1			2		5
	Design Applications					4				1		5
	Design Elements and Visual Principles		2		3		5		5	8		23
	Deep Learning and Image Enhancement		4	3	2	1			4	9		23
AGDIP	Al Modeling and Generative Techniques									3	3	6
	Computer Vision and Image Processing						2	2		8		12
	Interaction Design and User Experience						2	2		3	2	9
	Artificial Intelligence and Machine Learning Modeling						1	4		3	3	11
	Design Applications						2	1		1		4
	Design Elements and Visual Principles						2	6		2	4	14
	Visual Cognition and Attention							1		1	1	3
	Datasets & Resource Utilisation									5	1	6
	Optimization Algorithms & Generation Techniques						1	2			2	5
	Semantic and contextual applications							1			2	3
AIDAG	Layout Design & Automation										5	5
	Snimation & Graphics Generation										3	3
	Machine Learning & Design Automation										3	3
	Design Applications							2				2
	Datasets & Resource Utilisation							1				1
	Optimization Algorithms & Generation Techniques							1			3	4
	Semantic and contextual applications							2				2
AVAERM	Al Modeling and Generative Techniques								1	1		2
	Computer Vision and Image Processing		1							1		2
	Evaluation Modeling & Data Mining		1							1		2
	Design Language and Branding		2						2	1		5
	Visual Perception and Colour Analysis		3									3

Note: AI-driven design automation and generation = AIDAG; AI-assisted graphic design and image processing = AGDIP; AI in artistic and creative design processes = AACDP; AI-enhanced visual attention and emotional response modeling = AVAERM.

AIDAG: This paradigm leverages cutting-edge technologies like deep generative adversarial Networks to automate design processes and generate creative content. These networks are particularly adept at learning from a dataset and producing new, high-quality designs that are indistinguishable from human-generated work. Additionally, ant colony algorithms are used for optimization tasks within design layouts, enhancing the efficiency and creativity of automated design solutions.

AGDIP: Here, AI assists in refining graphic design elements through advanced image processing techniques. Conditional generative adversarial nets are employed to generate context-aware graphic elements that adapt to specific design requirements and user inputs. This paradigm also utilizes sentiment-specific word embeddings to infuse emotional intelligence into designs, allowing systems to understand and process user emotions and context more effectively.

AACDP: AI technologies like latent diffusion models are pivotal in this paradigm, enabling the creation of detailed and contextually relevant artworks by interpreting and materializing complex design briefs. These models support high levels of customization and creativity, facilitating unique artistic expressions that resonate with target audiences.

AVAERM: This paradigm focuses on optimizing designs based on how users perceive and emotionally react to them. It utilizes sophisticated models that combine convolutional neural networks, recurrent neural networks, and attention mechanisms to analyze and predict user responses. This integration allows for the design of more engaging and emotionally compelling graphic elements that capture and retain user attention effectively.

Within these four paradigms, we identified 17 distinct categories and their manifestations across different paradigms. Layout design & automation: occupies a core position in AIDAG, mainly used for optimizing and automating design layouts, such as posters and page layouts [49], enhancing the efficiency and quality of layouts [68]. Animation & graphics generation: Also falls under AIDAG, focusing on the technology for automatically generating animations and scalable vector graphics, reducing the workload [50]. Machine learning & design automation: covered in both AIDAG and AGDIP, leveraging machine learning to mimic the human brain's thought process to improve decision-making in design work [50]. Design applications: mentioned in AIDAG, AACDP, and AGDIP, reflecting the focus on the characteristics of design applications, usage frequency, and the enhancements in automation and intelligence [66]. Datasets & resource utilisation: Stressed in both AGDIP and AACDP, emphasizing the importance of utilizing and optimizing datasets in design projects [66]. Optimization algorithms & generation techniques: This category appears in both AIDAG and AGDIP, where technologies like the ant colony algorithm and conditional generative adversarial nets are used to improve the quality of automatically generated designs [49,50], creating complex and advanced design solutions [93]. Semantic and contextual applications: Highlighted in both AIDAG and AGDIP, for instance, employing Sentiment-specific word embedding and textual design concept, illustrating the attention to understanding content meaning and integrating sentiment in the design process [51], emphasizing the incorporation of emotion and meaning to create works with emotional resonance [94]. AI modeling and generative techniques: Extremely important in AGDIP and AVAERM, research has shifted from simple image technology to more advanced deep learning models and complex generative techniques since 2022 [52]. Computer vision and image processing: Involves applications in image recognition and processing [70], constituting a significant part of both AGDIP and AVAERM. Interaction design and user experience: Highlighted in both AGDIP and AACDP, moving from basic user interaction to deep involvement and cultural sensitivity shifts [95], predicting users' attention to images and integrating cultural elements to create new patterns [96]. Visual cognition and attention: In AGDIP, the combination of AI with visual attention enriches the user experience in graphic design [61,70]. Design elements and visual principles: This category is expanded in both AGDIP and AACDP, mainly assisting with the understanding and application of design elements such as color and contrast [71]. Moreover, progress in areas like layout, Text, and graphic design style demonstrates AI's delicate operation ability in handling details and shaping styles [54,65,71]. Deep learning and image enhancement: In AACDP, deep learning is used to improve image quality and visual effects, promoting design innovation and image optimization [55,65,77]. Evaluation modeling & data mining: In AVAERM, AI conducts design evaluation and data mining, used for analyzing market trends and optimizing user behavior [79]. By 2022, AI significantly contributed to design quality assessment, developing evaluation models that monitor performance and enhance design quality [58]. Design language and branding: In AVAERM, AI plays a crucial role in shaping and understanding design language and brand identity [79]. Visual perception and colour analysis: Research on AI's capability in understanding visual perception and conducting color analysis mainly reflects AI's role in refining and optimizing visual design elements in the AVAERM paradigm [79]. Artificial intelligence and machine learning modeling: In AGDIP, showing progress from basic models to focusing on attention mechanisms and recurrent neural networks, demonstrating advancements in simulating complex human visual and cognitive processes [53,56,67].

Each paradigm demonstrates its unique emphases and objectives, while also highlighting potential conflicts in technology application. The AIDAG paradigm first exemplifies AI's potential for enhancing design process efficiency and fostering innovation. It reflects the pursuit of increased design automation and high-quality output [49] through the optimization of design workflows and the automatic generation of design elements [50]. While the AGDIP paradigm focuses on specific technological applications for graphic design and image processing [70], its emphasis lies in the production of design elements, image enhancement [60,74]. On the other hand, The AACDP paradigm primarily explores AI's role in artistic and creative design processes, encompassing the application of machine learning and design automation, as well as the unique treatment of design principles and visual elements [63,71,75]. It highlights the potential of AI to foster design innovation, enhance artistic expression, and increase design personalization [54,65,71]. It emphasizes the potential of AI in fostering design innovation, enhancing artistic expression, and improving design personalization [58], offering emotional design solutions based on image segmentation, sentiment analysis, and understanding of design language and branding [79].

Conflicts exist between these paradigms in terms of technology and creativity, efficiency and personalization, as well as data-driven approaches and emotional understanding. The technological applications and automation tendencies of AIDAG and AGDIP could suppress creativity and emotional expression, whereas the focus of AACDP and AVAERM lies in maintaining the innovativeness and emotional connections of design. The efficiency brought about by automation might contradict the philosophy of pursuing personalized and customized designs, which requires more creative input and manual adjustments. Furthermore, a data-driven design approach might overlook the importance of human emotions and cultural diversity, clashing with design philosophies that emphasize artistic value and emotional connections.

### 3.4 Synthesis expression: theme reflection and construction

We observe a complementary and enhancing relationship between the four paradigms. For instance, the collaborative relationship between AIDAG and AGDIP reveals the complementarity of AI in the design process, where automated design generation offers preliminary schemes, and graphic design assistance refines these schemes. AACDP and AVAERM, on the other hand, place greater emphasis on the emotional resonance and cultural adaptability of design. These paradigms exhibit significant differences among themselves. AIDAG focuses on the automation and generation of the design process, emphasizing the enhancement of design efficiency and innovation through AI technology. AGDIP pays more attention to the auxiliary role of AI in graphic design and image processing. AACDP explores the role of AI in the artistic and creative design process, evolving from early genetic algorithms and evolutionary art exploration to the application of deep learning in design feature recognition and automation. Finally, AVAERM focuses on applications in enhancing visual attention and emotional response modeling.

Though these accounts underscore the potential for collaboration between AI technologies and human designers, they might not fully discuss the complexities of such collaboration in practice. For instance, the communication and collaborative process between designers and AI may encounter obstacles, such as technical barriers, differences in understanding, and changes in work habits [97]. Secondly, a too-optimistic view of AI technology's application in the design field might not take into account the potential risks and challenges [98], possibly exacerbating homogeneity in the graphic design industry, reducing originality and creativity [99,100]. Furthermore, the role of AI in design decisions might lead to the marginalization of designers' professional judgment [101]. Previous research has focused more on the functionality and effects of AI technology, paying less attention to discussions on the transformation of designers' roles, ethical responsibilities, and the technology's long-term impact on the design industry [102,103].

The synthesis reveals a new critical perspective: AI is not only a tool for achieving design automation and efficiency enhancements but also a medium for fostering innovation and deep human emotional exchanges. AI is redefined as both an assistant that helps designers accelerate iterations and improve techniques and as a medium that stimulates creativity and understands users' emotional needs. It prompts us to rethink the relationship process between design and designers: a comprehensive innovative process that integrates technology, art, and the humanities.

## 4 Discussion

### 4.1 Why is AI growing so rapidly in graphic design?

The rapid development of AI in the field of graphic design is primarily driven by advancements in technology, market demand, creativity, and practice. Among these, technological progress is particularly noteworthy. The swift advancement of deep learning, image recognition, natural language processing, and generative adversarial networks has significantly expanded the boundaries of graphic design. These advanced technologies not only make automation tasks possible but also empower machines to perform complex and creative design work.

The advanced nature of today's AI technology is not only reflected in the automation of routine design tasks but also in its ability to perform complex and innovative design work. For instance, Adobe Firefly leverages AI to quickly generate professional design variants based on text prompts, marking a significant leap in design efficiency and personalized customization [104]. Tools like Fotor AI and Designs.AI simplify the design process, enabling non-professional designers to create high-quality visual works, demonstrating AI's potential to lower the barriers to design [105]. In addition, AI technology also shows great potential in generating artworks and combining with NFT. AI design tools such as DALL-E, Midjourney and Stable Diffusion are not only capable of generating visual artworks based on textual prompts, but also of creating new types of artworks combined with NFT technology [106,107]. These tools are expanding the creative space for designers, allowing them to rapidly realize and iterate design concepts.

From the perspective of innovation drivers, AI technology offers designers a new array of tools for experimentation and exploring new creative directions, including style transfer, color matching, and graphic generation. For example, the use of neural networks for artistic graphic design and the automated layout design through the Play model [52,53], showcases how AI can mimic the creative process of human designers, understanding and addressing complex design problems. In design practice, design teams are increasingly adopting AI-assisted design tools. Adobe Sensei, for instance, not only optimizes workflows but also enhances team collaboration efficiency [58,79],It automatically generates multiple colour adjustment options for the image, allowing the designer to choose the result that best matches the emotional tone of the project. In addition, Sensei can provide customised design recommendations based on user feedback and design trend data to deeply understand and stimulate users' emotional needs. This not only accelerates the design process.

Additionally, tools like AutoDraw save valuable time for designers by automating the color palette process and the completion of design sketches, enabling them to focus on refining creativity and deepening concepts [108]. Fronty AI bridges the gap between graphic design and web design by converting images into useable HTML CSS code [109]. The Ming-style Furniture Midjourney Prompt (MFMP) toolkit, developed by the Midjourney Platform, can be used to develop and reproduce the aesthetic characteristics of Ming Dynasty furniture and to explore its application to modern furniture design [110]. And FlatMagic was an AI tool integrated into Photoshop that increased the productivity of professional comic book designers and expanded their creative space by automating the coloring process for black and white sketches [111].

Through these cases, we can see the practical application and results of AI in the graphic design industry. AI has not only improved the efficiency and quality of design but also provided designers with a broader space for creativity, making personalized and innovative design more feasible.

### 4.2 What are the current research trends and future directions in the field?

The integration of AI into graphic design is advancing an interdisciplinary fusion that not only enhances technical capabilities but also deepens the emotional and cultural resonance of design works. Here, we outline specific future research directions, supported by recent innovations and studies that exemplify the potential and benefits of AI in this domain.

Personalized design recommendations: future research should focus on developing AI systems that can analyze user interaction data in real-time and provide personalized design recommendations. For instance, a study demonstrated an AI system that adapts website layouts in real-time based on user behavior, significantly improving user engagement and satisfaction [112]. On this basis, researchers can explore how different AI interactions can affect human perceptual co-creation processes, providing design considerations for enhancing human-AI co-creation interactions [113].

AI in emotional response modeling: Integrating AI with emotional computing to create design tools that understand and respond to user emotions represents a significant leap forward. For example, A packaging design evaluation method based on Image Emotion Perception Computing (PDE-IEPC) to enhance the personalized emotional experience and effect prediction accuracy of design for an immersive and dynamic experience of human senses [114]. And, combining deep learning algorithms and CAD modeling optimization, the emotional tendencies and themes in advertisements are accurately extracted through sentiment analysis techniques to improve the emotional expression of advertisement art design [115].

Cultural adaptability in design: The ability of AI to adapt designs to various cultural contexts is an essential aspect of contemporary graphic design. One study uncovered colour association rules through data mining algorithms for extracting and applying colour resources with Chinese painting characteristics to the colour design of cultural creative products [116]. Future research could build on these foundations, exploring more sophisticated AI applications that can discern and incorporate deeper cultural narratives and historical elements into designs, thereby enhancing the global appeal and local relevance of design projects.

AI-Driven creative processes: exploring AI's role in automating and enhancing the creative process is a promising direction. For example, the CreativeConnect system, a tool that integrates a generative AI pipeline, facilitates the discovery of keywords and the generation of multiple design concepts by helping users to discover useful elements from reference images through keywords, displaying diverse restructuring options and sketch text descriptions [117].

By focusing on these specific areas, driven by concrete research examples, the field can move towards a more targeted and effective integration of AI technologies in graphic design. These directions not only demonstrate innovative applications but also address the practical benefits and challenges of implementing AI in real-world design scenarios.

### 4.3 A critical look at AI technology in graphic design

In the realm of graphic design, the application of AI technology is influenced by a dual impact of technological progress and market demand, as well as related to innovation drivers and the transformation of design practices. During this process, AI's limitations in understanding complex human emotions and aesthetics may hinder its ability to reach the level of artistic and emotional expression attainable by human designers [118]. At the same time, the pursuit of market demands may lead to the homogenization of design, and an over-reliance on data-driven design may overlook originality and creativity. The drive for innovation brings about technological and thought-process renovations; however, effectively integrating these new tools and ideas into the design process, ensuring they are widely understood, accepted, and successfully applied, remains a significant challenge.

Although most research discusses the fundamental changes AI brings to design, this technological trend may also introduce socio-economic issues such as professional ethics and reduced employment opportunities. Moreover, not all designers have equal access to AI technology. The high costs of technology and the barriers to technical knowledge may exclude certain groups, potentially exacerbating inequality within the design community, possibly leading to a new "technological divide." We find in AGDIP and AVAERM, especially, that data-driven design and emotional response analysis might involve the collection and processing of massive amounts of user data. This raises important issues regarding user privacy protection, transparency in data use, and security [119], which were not mentioned in the discussions above. Additionally, there was no in-depth exploration of how AI could handle and respect design needs within a globally diverse cultural background [120]. AI models' training data might possess cultural biases, potentially leading to design solutions that do not genuinely reflect the unique perspectives and needs of different cultural and social groups [121].

Overall, the application of AI technology in the field of graphic design is a complex and multi-dimensional phenomenon. Finding a balance, meeting market demands while maintaining the originality and diversity of design, and effectively combining human creativity with technological advancement, are key to driving the continuous development of the field of graphic design. This systematic review has certain limitations. It is based on a limited set of literature resources, which may not fully encompass all relevant studies and cases. Additionally, issues such as the adaptability of AI in diverse cultural contexts and the ethical responsibilities it entails have not been thoroughly explored.

### 4.4 The potential and opportunities of artificial intelligence in graphic design

**Fig. 6:**
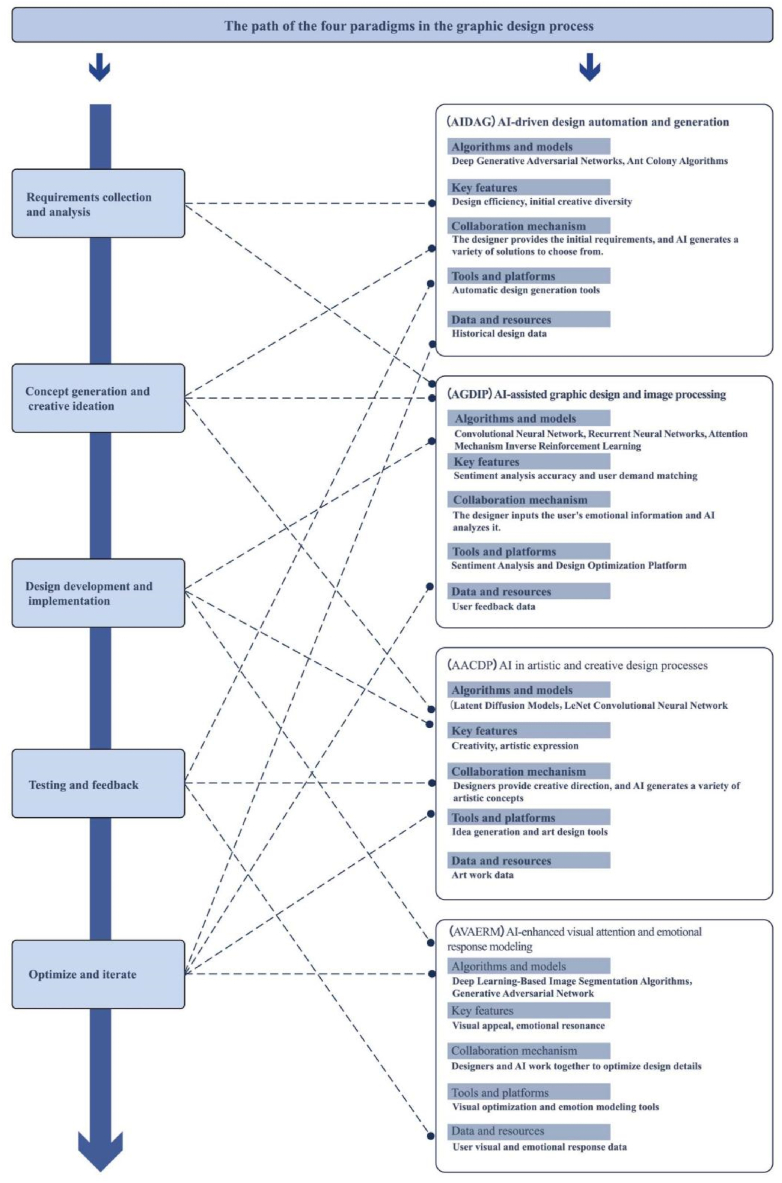
The path of the four paradigms in the graphic design process.

In this review paper, we systematically explore the revolutionary applications of AI within the field of graphic design and reveal the multidimensional potential of AI-designer collaboration through four innovative application paradigms, namely AIDAG, AGDIP, AACDP and AVAERM. We found that these four paradigms can enhance all stages of design. These paradigms demonstrate the diverse applications and profound impacts of AI technologies in the requirements gathering and analysis, concept generation and creative ideation, design development and implementation, testing and feedback, and optimization and iteration segments, respectively (as shown in Fig. 6).

First, in the requirements collection and analysis phase, the AIDAG paradigm automates the generation of diverse design solutions by leveraging advanced technologies such as deep generative adversarial networks and ant colony algorithms to provide designers with initial creative inspiration. Automation at this stage helps designers to quickly understand and integrate client requirements, improving the starting efficiency of the design process.

Entering the concept generation and creative conception stage, the AGDIP paradigm plays its role by accurately analysing user emotions and preferences through models such as convolutional neural networks and recursive neural networks, combined with sentiment analysis tools, to assist designers in incorporating more personalized elements into their creative conceptions. The accuracy of this sentiment analysis ensures that the design solution better matches the user's real needs.

In the design development and implementation phase, the AACDP paradigm uses techniques such as potential diffusion modeling to promote the deepening of creativity and artistic expression. the data-driven artistic innovation provided by the AI in this phase inspires designers to explore new visual languages and creative expressions, greatly enriching the creative level of design.

When entering the testing and feedback phase, the AVAERM paradigm optimizes design details through deep learning based on image segmentation algorithms and generative adversarial networks to enhance the visual appeal and emotional resonance of the design. This paradigm enables designers to fine-tune design elements based on users' visual and emotional feedback, ensuring that the final work can effectively touch users.

Finally, in the optimization and iteration phase, data and feedback from all four AI paradigms are recycled to continuously optimize and iterate the design solution. This process not only improves the overall quality of the design, but also enables the design solution to more accurately meet user needs, demonstrating the critical role of AI in the design optimization process.

Through this comprehensive perspective, we can see that AI technology not only provides effective support and enhancement at all stages of design, but also greatly improves the overall quality and innovation of graphic design through the in-depth integration of different paradigms. It provides an effective design path for future design work.

## 5 Conclusion

This paper provides an in-depth exploration of the current state and future trends of AI technology in graphic design using the critical interpretive synthesis approach. Through a systematic analysis of 33 papers, we identify and discuss four key paradigms: AIDAG, AGDIP, AACDP, AVAERM, and constructs an application path framework in the graphic design process, providing effective design paths for future design workflows and opening different perspectives for design practice. Future research should delve deeper into these topics to achieve a more comprehensive understanding. The application of AI technology in graphic design represents a complex and multidimensional phenomenon that not only alters the production process but also extends the boundaries of design. However, there are limitations to AI technology in understanding and simulating human emotions and aesthetics. Market demands may lead to design homogenization, presenting a significant challenge that requires attention and resolution.

## CRediT authorship contribution statement

**Hong Li:** Writing – review & editing, Writing – original draft, Methodology, Data curation, Conceptualization. **Tao Xue:** Visualization, Data curation. **Aijia Zhang:** Methodology, Data curation. **Xuexing Luo:** Methodology. **Lingqi Kong:** Supervision, Project administration, Conceptualization. **Guanghui Huang:** Supervision, Project administration, Conceptualization.

## Data and code availability statement

No data was used for the research described in the article.

## Funding

This work was supported by the Macau University of Science and Technology's Faculty Research Grant (No: FRG-24-049-FA).

## Declaration of Competing Interest

The authors declare that they have no known competing financial interests or personal relationships that could have appeared to influence the work reported in this paper.
